# '20 days protected learning' - students' experiences of an overseas nurses programme - 4 years on: a retrospective survey

**DOI:** 10.1186/1472-6955-10-7

**Published:** 2011-04-19

**Authors:** Gill Jordan, Petra Brown

**Affiliations:** 1 School of Health and Social Care, Bournemouth University, Bournemouth House, Christchurch Road, Bournemouth BH1 3LH. UK

## Abstract

**Background:**

From September 2005 the Nursing and Midwifery Council (NMC) introduced new arrangements for the registration of non-EU overseas nurses which requires all applicants to undertake '20 days of protected learning' time in the UK and for some, a period of supervised practice. A survey was undertaken at Bournemouth University, which offers a '20 days protected learning only' programme, to elicit overseas nurses' demographic details, experiences in completing the programme and their 'final destinations' once registered.

**Methods:**

An online survey was devised which contained a mixture of tick box and open ended questions which covered demographic details, views on the programme and final destinations This was uploaded to http://www.surveymonkey.com/ and sent out to nurses who had completed the Overseas Nurses Programme (ONP) with Bournemouth University (n = 1050). Quantitative data were analysed using descriptive statistics and the qualitative data were coded and analysed using content analysis.

**Results:**

There were 251 respondents (27.7% response rate). The typical 'profile' of a nurse who responded to the survey was female, aged 25-40 years and had been qualified for more than 5 years with a bachelors degree. The majority came from Australia on a 2 year working holiday visa and the key final destination in the UK, on registration with the NMC, was working for an agency.

There were five key findings regarding experience of the programme. Of those surveyed 61.2% did not feel it necessary to undergo an ONP; 71.6% felt that they should be able to complete the programme on-line in their own country; 64.2% that the ONP should only contain information about delivery of healthcare in UK and Legal and professional (NMC) issues; 57% that European nurses should also undergo the same programme and sit an IELTS test; and 68.2% that the programme was too theory orientated; and should have links to practice (21%).

**Conclusions:**

The NMC set the admissions criteria for entry to the register and Standards for an ONP. The findings of this survey raise issues regarding the perceived value and use of this approach for overseas nurses, and it may be helpful to take this into account when considering future policy.

## Background

The Nursing and Midwifery Council (NMC) introduced new arrangements for the registration of non-EU overseas nurses in September 2005. This required all applicants to undertake '20 days of protected learning' time in the UK and for some, a period of supervised practice, to ensure that all applicants are fit to practice prior to entering the NMC register [1,2]

A literature review in 2010 revealed that there were no articles or papers specifically written about nurses undertaking the '20 days protected study only' course. Related literature available included Buchan et al's [3] study of the international recruitment of nurses which included those from Australia, New Zealand, USA and South Africa. However, this study was published prior the the NMC mandatory requirements [1] for overseas nurses wishing to register with the NMC.

There was literature pertaining to the Supervised Practice Overseas Nurses Programme, although much of this was about Adaptation Programmes, prior to the NMC's 2005 revision of how nurses from overseas could join their register. There were large scale active international recruitment strategies in both the NHS and Independent sectors during the years 2002/2003, and many were reported on. The areas discussed in this literature were mainly centred on the cultural issues of overseas nurses from non 'old' commonwealth countries [3-9] and the outflow of experienced nurses from developing countries [10,11].

The lack of literature related to a '20 days protected learning only' course could be attributed to the high numbers undertaking the course with Bournemouth University, who have only recently undertaken this survey. Data from the NMC [12] and our own enrolment data suggest that 80% of all nurses from Australia, New Zealand, Canada, South Africa and United States of America who have undergone this type of overseas nurses programme, did so with Bournemouth University.

Bournemouth University only offers a '20 days protected learning only programme'. The great majority (94.4%) of participating nurses come from Australia, New Zealand, Canada, South Africa and United States of America, as the NMC [1] (on the whole) do not require nurses from these countries to undertake the Supervised Practice Overseas Nurses Programme. Our programme has been running since April 2006 and has been accessed by over 1300 nurses, and the delivery and content has essentially not changed in the past four years. The Bournemouth University ONP is delivered via a flexible learning route whereby nurses are required to successfully complete a distance learning study guide and pass a multiple choice test. The study guide follows the NMC learning outcomes [1,2], and is supported by three contact days, which all amounts to the equivalent of 150 hours (20 days) study. The majority of the activities direct nurses to websites for their answers, although some activities are of a more reflective nature. The rationale for the use of a study guide is that nurses can study in their own environment and in their own time, and that the study guide would be a good resource for further study. The use of websites encourages familiarisation with UK sites as well as enabling nurses to study without the use of a conventional library. Support for this is via email. The programme is not credit rated as, on the whole, the participating nurses are at degree level and are only interested in gaining NMC registration. A small survey was undertaken in 2007 [13] where 343 nurses were asked to complete a questionnaire of their experience of the Bournemouth programme which elicited a 13.4% (n = 46) response rate when the programme was in its infancy. Many of the responses reflected the nurses' views of the NMC regulations for attaining registration, rather than the delivery of the actual programme. The overall outcome was that 80% of nurses surveyed felt that they did not need to undertake an Overseas Nurses Programme, and with this in mind the ONP team at Bournemouth University wanted to explore whether the same views were still held by more recent participants.

### Aim of the survey

i) To elicit demographic details of nurses undertaking an Overseas Nurses Programme to understand who was taking this programme

ii) To explore the experiences of nurses undertaking an overseas nurses programme in terms of the learning outcomes specified by the Nursing and Midwifery Council [1,2]

iii) To obtain information about the 'final destinations' of nurses in order to explore their impact within the UK workforce

## Methods

### Study Design and Setting

The study was a descriptive cross-sectional survey of nurses who had undergone an Overseas Nurses Programme with Bournemouth University from 2007-2010 using a web-based survey tool.

### Study population and questionnaire distribution

The target population was all 1051 nurses who had enrolled on the programme since the previous survey in April 2007 up to January 2010. Selective sampling was not undertaken and all students were given the opportunity to take part to maximise demographic variability, response rates and generalisability [14]. As the NMC requirements [1,2] and content of the Bournemouth course had not changed over the period 2007-2010, the decision was made not to split the participants into their specific yearly intakes.

An online survey was devised which contained a mixture of tick box and open ended questions (Additional file 1). This was piloted by team members to enable assessment of accuracy, logical flow and ease of use. The questionnaire took a maximum of 15 minutes to complete which was felt to be a reasonable time frame; time for completion being crucial in affecting response rates [15].

The questionnaire was uploaded to http://www.surveymonkey.com/ as the software is easy to navigate and user friendly, which has been shown to improve response rates [16]. It also allowed easy access for students by including the web link in the invitation email. Email contact was used rather than course enrolment addresses as this has been shown to be an effective way of quickly and easily reaching a wide population of students [16,17]. This was particularly relevant as we had no way of knowing whether the overseas nurses were still residing in the UK, travelling, or had returned to their home countries as there was a time lapse between the nurses completing the programme and the survey. In order to respect the autonomy of the survey participants and the need for implied consent, participants were given an overview and information sheet about the intended research [18]. Participants were informed that by completing the questionnaire, they would be giving implied consent to participate in the research [19].

#### Questionnaire Design

Questionnaire items were developed from a content analysis [20] of feedback received from student course evaluations on the last day of the course, data obtained from the previous survey, and a desire to explore if overseas nurses were having an impact on the UK nursing workforce.

The questionnaire comprised 22 items with domains including personal demographics, professional qualifications, employment history in the UK and the Overseas Nursing Programme itself. There were 17 closed questions and 5 open questions. The aim of the qualitative open-ended questions was to add dimension to the closed questions as it was was impossible to envisage all the factors which might have influenced the nurses' experiences [20].

### Data Entry and Analysis

All results were downloaded from the http://www.surveymonkey.com/ website. Quantitative data were analysed using descriptive statistics and the qualitative data were coded and analysed using content analysis where comments related to each question were coded, grouped and similarities and differences noted [20].

## Results

### Response rate

Ten nurses (response rate 1.1%) replied after an initial email. This increased to 251 nurses (27.7% response rate) following a second email. One hundred and forty-five (13.8%) emails 'bounced' leaving 72.3% (n = 654) receiving the invitation email. Although disappointing this is representative of on-line surveys [21]. Despite 27.7% being a low response rate, data from the NMC [12] and our enrolment data suggest that Bournemouth University facilitated 80% of all nurses undertaking a '20 days protected learning' ONP from Australia, New Zealand, Canada, South Africa and United States of America. This could be seen as increasing generalisability of the findings.

### Demographic Information

**Figure 1:**
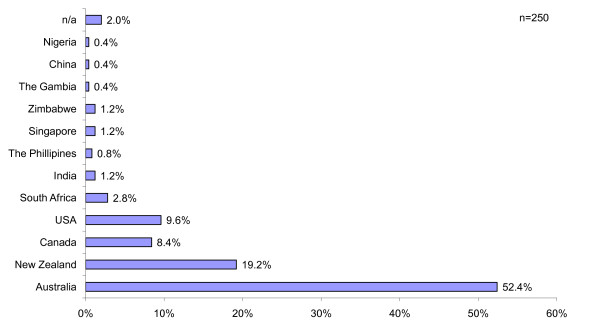
Country of pre-registration nurse qualification

Females accounted for 92.4% of those surveyed. Most nurses were within the 25-40 years age group (69.9%) with 8.8% being under 25, and 21.3% over 40 years. The vast majority obtained their pre-registration nursing qualification in Australia (52.4%), with New Zealand (19.2%), Canada (8.4%) and the United States (9.6%) comprising the highest numbers from other countries (Figure 1).

Most nurses (52.4%) had been qualified for more than 5 years before undertaking their ONP with 41.6% being qualified between 2 and 5 years. Only 6% had been qualified for less than 2 years which is explained by the NMC regulations [2] which state applicants must have been practicing as a registered nurse for at least 12 months after qualifying before they can apply to join the register. The registration process can take anything up to one year to complete - hence nurses being qualified for two years before actually working in the UK.

**Figure 2:**
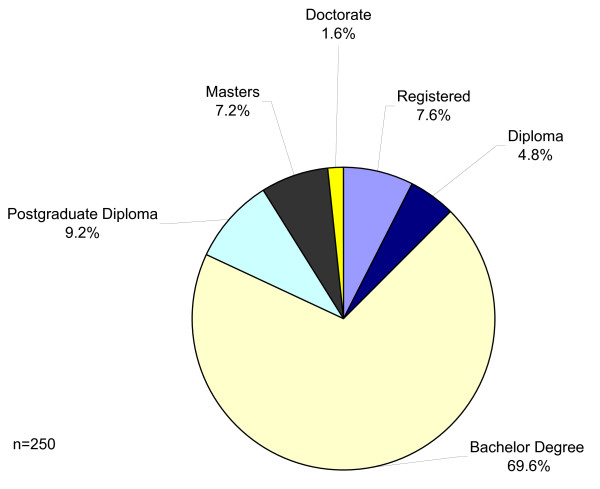
Level of academic study on admission to ONP

In line with the pre-registration nursing programmes of most of the countries represented in the survey, 69.6% of nurses had studied to first degree level. Those who studied at a higher academic level numbered 18%, whilst only 12.4% either gained a diploma or undertook a pre-registration course without academic credit (Figure 2).

Of the nurses surveyed, 53.4% (n = 134) gave information about the post registration nursing courses they had completed. This resulted in 49% (n = 63) having completed courses in critical care subjects (critical care, theatres, trauma or endoscopy); 20% (n = 26) in Medical care areas (palliative care, diabetes, elderly, health sciences and continence); 9% (n = 12) having qualified as midwives in addition to their nursing qualification. The remainder had undertaken paediatric, mental health or non-clinical courses.

#### Working in the United Kingdom

**Figure 3:**
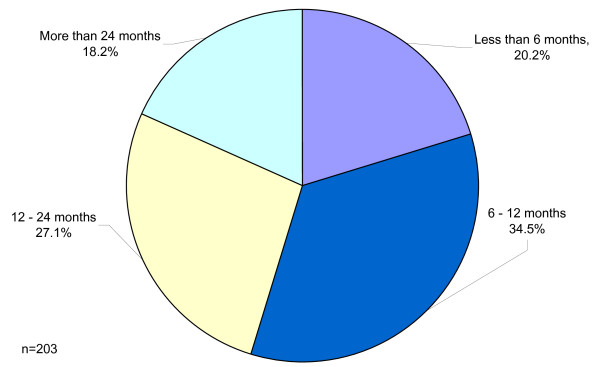
Length of time working in the UK

Nurses were asked how long they had worked in the UK since registration. The response to this was 81% (n = 203). Of these responses, it was interesting to note that the answers corresponded with the length of time visa restrictions allowed them to stay in the country. Most nurses had a 2 year working holiday visa and intended to work intermittently during their 2 years in the country. Others had emigrated, whilst others (noticeably those from USA) had 5 year visas (Figure 3). However as the survey spanned 3 years some nurses had returned to their own countries. Equally some nurses had only recently completed their ONP and so had been working for a shorter time when the survey was completed.

Additionally nurses were asked about their recent employment history. Overall 49.3% had worked as a registered nurse within the UK at some time. Of those remaining, 36.7% were working as a registered nurse in their home country, 8% were not working as a registered nurse, 4% were travelling, 1.2% were undertaking an academic course and 0.8% were working in the EU.

**Figure 4:**
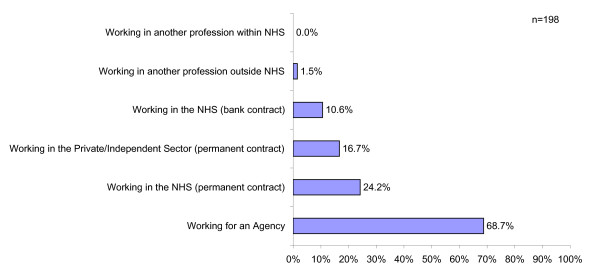
Areas of work within UK healthcare services

Nurses who were working/had worked in the UK was examined more fully. The reported findings (Figure 4) showed that most had worked for nursing agencies (68.7%), although 24% had had permanent contracts within the National Health Service, 16.7% had been working in the independent sector and 10.6% had worked for the NHS on bank contracts. The remaining 1.5% had jobs in another profession.

Thus it could be argued that overseas nurses from Australia, New Zealand, Canada, USA and South Africa do not have an impact on the NHS workforce in terms of taking up permanent jobs, rather they support the NHS staffing shortages via agency placements.

#### NMC Overseas Nurses Programme

**Figure 5:**
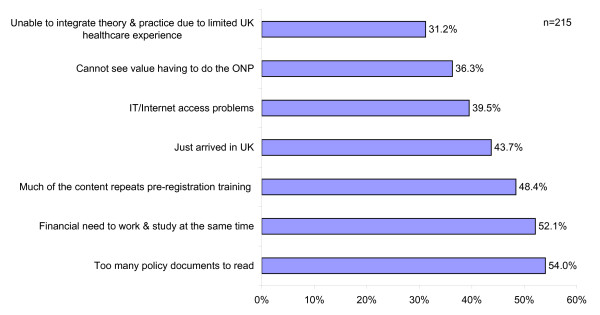
Main barriers to learning

Nurses were asked about any barriers they felt affected their learning whilst undertaking their ONP - the results are in Figure 5.

The main barriers were: too many policy documents to read (54%), financial need to work and study at the same time (52.1%), much of the content repeating their pre-registration training (48.4%), the fact that they had only just arrived in the UK (43.7%). IT/Internet access problems (39.5%), their not seeing value in doing an ONP (36.3%) and their inability to integrate theory with practice as they had no or limited experience of UK healthcare were cited as other reasons. Additional barriers to learning that were explored in the list included: lack of permanent accommodation (25.6%), the desire to complete the programme in 20 days (23.3%), lack of peer support (21.4%), Isolation and culture shock (14.9%), lack of IT skills (1.4%), and the fact that it did not suit their learning style (7.4%).

Analysis of open ended answers which accompanied this question emphasised that information requested in the activities was a repeat of their pre-registration programmes, and that completing an ONP did not 'identify if I was a good/safe nurse or not'. Many felt that the NMC content was not appropriate in that they had studied topics such as Health promotion, communication, evidence-based learning, medicine calculations and interprofessional practice within their own country's curriculum to join their professional register. They also highlighted that multi-culturalism was the same in their own countries

Their comments included:

'I don't feel I have learned a lot from my ONP - most of the information I have already covered in my degree back home'

'I do not believe the ONP helped me in my practice; it was my original undergraduate course that enabled me to learn the skills I use today'

Additionally, the majority of nurses (71.6%) stated that they would have completed the ONP study guide in their own country given the choice, and cited financial and social reasons for this.

'this course should be completed online in your home country so you are able to work as a registered nurses as soon as you arrive in the UK'

'it would be much better if the course could be done at home where you aren't stressed about finances and living arrangements'

'not being able to work and taking 4 weeks off when the course was expensive. I lost valuable time and money doing the course'

'course fees on top of other costs (IELTS, NMC fees etc.) contributes to many Australians not registering as nurses'.

Interestingly 24.7% of nurses commented on the benefit of undertaking supernumerary days in practice within their open ended answers. The feeling that the course should integrate theory and practice under the guidance of a mentor in a short clinical placement was strong, but that this could be optional. Comments which summed up many nurses' feelings included:

'I think some supernumerary days in practice would have been fantastic as I had not set foot in a UK hospital when I did the course'

'strongly feel that supernumerary days would have been helpful for me working as an RN for the first time in UK'

Overall, only 8.8% of nurses felt that completion of the ONP had a significant impact on their practice, while 33.5% stated 'partially', and 47.6% believed it had no impact at all (10.1% of nurses had not worked since completing the course). In terms of the NMC content nurses were asked which areas had had a significant impact on their practice as an overseas nurse. The main subjects they felt helpful were the NMC and Legal related subjects (67.2%) and delivery of Healthcare in the UK (52.6%). Australian nurses in particular felt that the standards and 'systems' of the UK and Australia were so similar that they did not need to undertake any type of ONP. Other subjects that nurses would have liked included was information about seeking employment in the UK (42.7%), career development in the UK (50.2%), nursing procedures (39.4%), specialist roles in nursing (35.2%) and clinical skills (28.6%).

'Much of the content prescribed by the NMC was irrelevant, as there is little difference to my home country. The only areas that needed to be taught were the structure and the function of the healthcare system in the UK, and the legal and professional responsibilities of nurses in the UK'

'I felt a booklet to read and a lecture on policies, the NHS and NMC only would be more beneficial and less time consuming'

**Figure 6:**
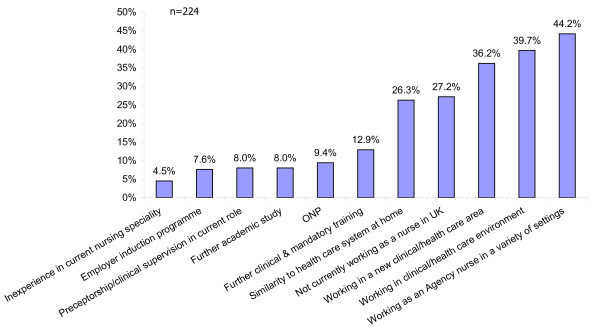
Other factors impacting practice in the UK

Nurses were asked to comment on other/any factors which had impacted their practice as a Registered Nurse in the UK. These are recorded in Figure 6. Completion of an ONP only gained 9.4% of their responses, with working as an agency nurse in a variety of settings (44.2%) and actual working in clinical/healthcare environment (39.7%) contributing most highly to their experiences - they felt 'on the job' training would have served their learning needs better than an ONP. Their comments included:

'I would have adapted and learnt the UK system while working'

'because we are already working qualified professionals, and since the policies and procedures change from hospital to hospital it may have been quicker, easier and cheaper to learn about the system while working in the system'

'everything I have learnt is from working in the NHS'

The final question asked whether nurses felt it was necessary to complete an ONP in order to work as a Registered Nurse in the UK. This resulted in 61.2% answering No, while 38.8% answered Yes. There were many comments from all nationalities of nurses which accompanied this question. The majority felt they had had adequate preparation in their own country to work as a Registered Nurse in the UK, although some acknowledged that the delivery of healthcare in the UK was different in some aspects. The majority (61.2%) felt they had not benefited from undertaking an ONP, and that a theoretical course did not assess their nursing abilities and competence:

'It did not teach me anything that would have a direct impact on my nursing'

'now that I am in the workplace, I must say that very little of what we covered helped me with the transition into my NHS staff nurse job'

'I felt that some sections of the ONP were almost patronising as I have a lot of experience working with people from different cultures'

'Too much theoretical work when a lot of nursing is about practice work'

However, 38.8% of nurses felt there was a need for some sort of Overseas Nurses programme with comments such as:

'I truly believe that the ONP standardises the experience for all foreign nurses and does benefit us. The benefit might not be recognised right away at the end of the course, but the true value comes once you are in the workplace and you have a better understanding of where you fit in'

'roles and responsibilities slightly different, terminology different, ONP familiarises overseas nurses with this'

'as an overseas trained nurse I feel it was necessary to complete ONP because it introduced me to nursing policies and let me know what is expected of me as a nurse in the UK'

There was much comment about European Nurses (particularly from those who had worked alongside nurses from Europe), and their exemption from an ONP, and in particular the language assessment. Open ended answers to this question in the survey elicited some very heated responses:

'I felt I was discriminated against by having to do the ONP when EU nurses don't have to do it'

'it is ridiculous that being Australian doesn't mean you can work as a nurse in the UK, but if you are from Europe you can - even if you can barely speak English!'

'I found it EXTREMELY unhelpful that as an Australian, who completed my degree in English, that I had to complete an IELTS test - there should be different entry criteria for those who have clearly been speaking English all their lives'

## Discussion

The original aim of the research was the discovery and exploration of overseas nurses' views regarding the NMC's requirement to undertake an ONP and to obtain information about the 'profile' and 'final destinations' of nurses who had undergone an ONP with Bournemouth University.

The typical 'profile' of a nurse who responded to the survey was: Female, aged 25-40 years and qualified for more than 5 years with a bachelors degree. The most common specialty they had previously worked in was the critical care arena with the majority coming from Australia on a 2 year working holiday. However this 'profile' does not necessarily reflect the whole population in the programme as many people who did not respond were under 25 years had been qualified under 5 years, and so may have raised different issues. The low response rate from this group could be explained by them travelling and unable to access emails as most were on working holidays.

In response to the question of whether overseas nurses from Australia, New Zealand, South Africa, Canada and the United States of America felt it necessary to undertake an overseas Nurses Programme, 61.2% believed it wasn't. However, this percentage has decreased from a much smaller survey undertaken in 2006 where the same question was asked and 80% believed it was not necessary. The high number in the 2006 findings could be explained by the NMC regulations only being in place for a year at the time and many respondents knew colleagues who had travelled to the UK before then and didn't need to undertake an ONP to register as a nurse.

The main theme of nurses was they believed they should not have to undertake an Overseas Nurses Programme as having a Bachelor of Nursing qualification should be considered adequate for NMC registration, and that the NMC Standards did not address subjects which were needed for *clinical *nursing in the UK.

The third of nurses who felt there was a need for some sort of ONP felt the current format was too detailed with some subjects, either not relevant, or a repetition of topics they were already familiar with. Given the graduate status and further academic training of the nurses surveyed, it is not surprising that they felt that they were repeating their pre-registration training. The main areas they felt required some 'theoretical' input were surrounding the delivery of healthcare in the UK and the legal and professional (NMC) issues associated with it. Although recognising that NMC may be looking for standardisation of learning outcomes across all registrants, this could be something to be considered in the future.

It was interesting to note that, although not stipulated by the NMC, some nurses favoured the opportunity for an optional clinical placement within the programme under the guidance of a mentor. They felt it would help them integrate into clinical practice in the UK more easily, which they deemed more important than the theory elements of the programme. If this was to be considered it would have logistical implications for Bournemouth University as clinical placements are already at a premium for pre-registration students, and additional numbers would be difficult to accommodate.

Many nurses believed that they should have been allowed to undertake the distance learning course in their own country on-line. This would have saved much time when they arrived in the UK when they were securing accommodation and employment to earn money. They did not feel studying the programme actually in the UK, where the values and culture were so similar to their own, made any difference to their integration into UK nursing and suggested a system similar the NCLEX-RN exam required to practice nursing in the USA [22] should be available. Nurses sitting the NCLEX-RN can do this in their home country (i.e. Canada, Australia, New Zealand and South Africa) before entering the USA, so a like process could alleviate many of their issues regarding lack of peer support and financial pressures when first arriving in the country.

Delivering the ONP on-line would prove a challenge with the large numbers accessing the programme in tracking their completion times, not to mention marking 100 activities per nurse electronically! Currently students write the answers within their study guide which controls the 'cut and paste' element of on-line work. The NMC [1,2] clearly state that nurses undertaking the ONP have to be resident in the UK at the time, hence the contact days which are used not only to support the nurses, but as a 'check' that they are in this country.

With respect to the nurses' views on having to undertake an IELTS exam, many felt this was not necessary and an additional expense. Regulations for other international students studying at Bournemouth University state only those whose first language is not English have to attain a satisfactory pass in an IELTS exam. The NMC Register application form asks nurses to state which language their initial training (with degree) was taught in, and as all nurses in the survey had stated 'English', they felt undertaking an IELTS exam was unnecessary.

Generally, once students had accepted they had to undertake an ONP, they related the benefits of meeting other students and in many cases reported making lasting new friends. They appreciated discussing issues of working and living in the UK with others in a similar position, but a major anxiety was lack of ability to earn money whilst studying.

In respect of final destinations, at the time of the survey four-fifths of nurses had used their registration to work in the UK. The majority had worked for agencies rather than taken contracts within the NHS as they wanted the flexibility of choosing when and where they worked. This reflects the principle of a working holiday which was the reason for most nurses entering the UK.

### Strengths and Limitations of the survey

The survey targeted all the nurses who had undertaken an ONP with Bournemouth University over 3 years and achieved a response rate of 27.7%. Although this was disappointing this is representative of on-line surveys [21], and included nurses from a wide variety of nursing backgrounds and countries. Future research on this topic must address the low response rate and methods of improving it [12]. The survey was only undertaken in one university, but considering Bournemouth University prepares the greatest number of Overseas Nurses undertaking the '20 days protected learning' programme for entry to the NMC Register, this can be seen as capturing the majority view. No other survey of this kind has been undertaken, so this study provides data to inform a future policy and practice in this area.

## Conclusion

This survey recognises nurses' views of their ONP. Most were Female, aged 25-40 years and qualified for more than 5 years with a bachelor's degree. The most common specialty they worked in was the critical care arena, with the majority coming from Australia on a 2 year working holiday visa.

Only just over one third felt there was a need to undertake an ONP. Of the remainder many felt it should only include the topics of Delivery of the Health Service in the UK and Legal and Professional (NMC) Issues. Overall only a very small number of nurses (8.6%) felt that the overseas nurses programme had a significant impact on their current nursing practice in the UK, with 33.5% answering 'partially' and 47.6% saying it had no impact at all. They indicated that employer mandatory and induction training had a greater impact on their subsequent practice.

Criticism of the programme was mainly directed at the NMC's need for nurses to undertake the programme and the NMC prescribed learning outcomes. Most nurses felt that had a wealth of post registration academic and specialised clinical nursing experience. They felt this was not recognised by the NMC application process, which appeared only to consider their pre-registration training. When asked if there were any other topics they felt should be included in the programme, nurses indicated a need for information on seeking employment in the UK and UK career development. Over one third of the nurses also indicated a desire for more information on clinical procedures. It would be fair to say that the distance learning structure was well evaluated by students who preferred it to having 20 days taught content in a classroom.

In terms of final destinations, the majority had worked for agencies rather than taken contracts within the NHS as they wanted the flexibility of choosing when and where they worked. This reflects the principle of a working holiday which was the reason for most nurses entering the UK. Hence it could be argued that overseas nurses from Australia, New Zealand, Canada, USA and South Africa do not have an impact on the NHS workforce in terms of taking up permanent jobs, rather they support the NHS staffing shortages via agency placements.

## Competing interests

The authors declare that they have no competing interests.

## Authors' contributions

GJ and PB were responsible for the study conception, design, data collection and data analysis. Both authors read and approved the final manuscript.

## Pre-publication history

The pre-publication history for this paper can be accessed here:

http://www.biomedcentral.com/1472-6955/10/7/prepub

## Supplementary Material

Additional file 1 ONP Nurses survey questionnaire Click here for file
