# Supplementary material for: '20 days protected learning' - students' experiences of an overseas nurses programme - 4 years on: a retrospective survey
Source: BMC Nurs. 2011 Apr 19;10:7. doi: 10.1186/1472-6955-10-7 (PMC3111357; doi:10.1186/1472-6955-10-7)
Supplement: Additional file 1 — ONP Nurses survey questionnaire [file 1472-6955-10-7-S1.DOC]

## ONP ’20 days protected learning’ Survey Questionnaire (January 2010)

1. Gender

Male

Female

2. Age

< 25

25-40

>40

3. Nationality

Open box

4. Country of Pre-Registration Nurse Education

Open box

5. How long have you been a Registered Nurse in your home country?

< 2 years

2-5 years

> 5 years

6. What is the highest academic qualification you have obtained?

Registered Nurse/Midwife-Certificate

Diploma

Bachelor Degree

Post graduate Diplomaate Certificate/Diploma

Masters egree

Doctorate

7. Please list any speciality post registration nursing/midwifery courses you have completed. (E.g. Critical Care Oncology course etc)

Open box

8. Where are you working currently?

Working as a registered nurse in UK

Have worked in UK as a nurse

If yes to either of these questions, please go to question 9

If no, please go to question 10

Working as a registered nurse in Europe

Working as a registered nurse in your home country

Not working as a registered nurse

Travelling since completing ONP but intending to return to UK to work as a nurse

Undertaking a full time academic course

Not working

Other (please specify) ->Open box

9. If you are or have worked as a Registered Nurse in the UK, what are / were your employment details?

Working in the NHS (permanent contract)

Working in the NHS (bank contract)

Working for an Agency

Working in the Private/Independent Sector (permanent contract)

Working in another profession within NHS

Working in another profession outside NHS

10. Length of time working in UK as a Registered Nurse

< 6 months

6 - 12 months

12 months – 24 months

> 24 months

11. Course evaluations have shown us that there are BARRIERS TO LEARNING whilst completing the Bournemouth University ONP. Which of the following do you agree with?

Just arrived in UK

Isolation & Culture shock

Lack of peer support

Lack of permanent accommodation

Financial need to work & study at the same time

Need to complete ONP in a month

IT/Internet access problems

Lack of IT skills

Does not suit learning style

Too many policy documents to read

Much of the content repeats pre-registration training overseas

Unable to integrate theory & practice due to limited/no UK healthcare experience

Cannot see value having to do the ONP

12. Please give details of any other barriers you have experienced whilst studying the ONP with Bournemouth University.

Open box

13. Some students would like the addition of / different LEARNING STRATEGIES to help them assimilate all the required information. Which of the following do you agree with?

Completion of study guide in home country

More taught days with lecturers

More face to face contact with lecturers

Lecturer telephone support

Web based discussion forum/chat room to link up with other students

Printed copies of resources required to complete workbook

CD-Rom copies of resources required to complete workbook

Typed rather than handwritten answers

Supernumerary day/s in practice to experience UK health care delivery

A higher academic level of study

14. Some students would also like more information on topics not necessarily required to meet the NMC/ONP learning outcomes. Would you have found it useful to have information on the following?

Clinical skills

Nursing procedures

Specialist nursing roles

Career development in the UK

Post graduate study in the UK

Cultural adaptation to the UK

Professional socialisation issues

Seeking employment in the UK

Nothing else is needed

15. Which of the NMC prescribed sections in the workbook do you consider has had significant impact on your practice as an overseas nurse?

UK as a multicultural society

UK Health Care Provision

Changing roles in nursing

Quality Assurance

NMC Code of Professional Conduct

Accountability Legal & Ethical issues

Evidence based practice & reflection

Mentoring preceptorship & clinical supervision

UK Nurse Education & development

PREP, Portfolios & profiles

Health Promotion/Education

Multi-disciplinary team working

Medicine administration

Numeracy skills

16. On reflection do you feel overall that the ONP has had a significant impact on your current nursing practice in the UK?

Yes

Partially

No

Not worked as a nurse

Other (please specify) - > Open box

17. Please feel free to provide more details of how the ONP has/has not had a significant impact on your current nursing practice

Open box

18. Given that working in the UK will have some impact on your nursing practice which of the following have had a SIGNIFICANT impact?

ONP

Working in clinical/health care environment

Working in a new clinical/health care area

Working as an Agency nurse in a variety of settings

Inexperience in current nursing speciality

Similarity to health care system at home

Employer induction programme

Preceptorship/clinical supervision in current role

Further clinical & mandatory training

Further academic study

Not currently working as a nurse in UK

19. Are there any other factors which have had a significant impact on your nursing practice since completing the ONP?

Open box

20. Do you feel that it was necessary for you to complete an ONP in order to nurse in the UK?

Yes

No

21. Why? (Note any further aspects not already covered by the questionnaire)

Open box

22. Please feel free to add comments on ANY aspects of the ONP that you feel have been important to you and not addressed by any of the questions.

Open box
